# Predicting surgical factors for unplanned overnight admission in ambulatory arthroscopic surgery of the knee: a prospective cohort in one hundred and eighty four patients

**DOI:** 10.1007/s00264-022-05436-8

**Published:** 2022-05-17

**Authors:** Nadhaporn Saengpetch, Ratthapoom Watcharopas, Chusak Kujkunasathian, Chalermchai Limitloahaphan, Chatchawan Lertbutsayanukul, Chaiyanun Vijittrakarnrung, Paphon Sa-ngasoongsong, Vanlapa Arnuntasupakul, Lisa Sangkum

**Affiliations:** 1 Department of Orthopedics, Faculty of Medicine Ramathibodi Hospital, Mahidol University, Bangkok, 10400 Thailand; 2 Department of Anesthesiology, Faculty of Medicine Ramathibodi Hospital, Mahidol University, Bangkok, 10400 Thailand

**Keywords:** Unplanned overnight admission, Ambulatory knee arthroscopic surgery, Clinical risk factor, Surgical invasiveness, Tourniquet time

## Abstract

**Purpose:**

Unplanned overnight admission (UOA) is an important indicator for quality of care with ambulatory knee arthroscopic surgery (AKAS). However, few studies have explored the factors related to the UOA and how to predict UOA after AKAS. This study aimed to evaluate the effectiveness of a standardized peri-operative protocol for the AKAS and identify whether a correlation exists between the peri-operative surgical factors and UOA in the patients undergoing AKAS. We hypothesized that more surgical invasiveness and prolong tourniquet time increase the risk of UOA after AKAS.

**Method:**

A prospective cohort study was conducted between October 2017 and March 2021. All 184 patients operated on standard AKAS protocol. The UOA is defined as overnight hospitalization of a patient undergoing AKAS. Demographic and peri-operative data were recorded, and the procedure was categorized based on the surgical invasiveness based on less invasive (intra-articular soft tissue surgery) (*n* = 65) and more complex surgery (involving extra-articular soft tissue surgery or ligamentous reconstruction) (*n* = 119). The clinical risk factors for UOA were identified and analyzed with multivariate analysis.

**Results:**

The incidence of UOA in the more complex group (*n* = 7, 14.3%) was significantly higher than in the less invasive group (*n* = 3, 4.6%) (*p* = 0.049). The peri-operative factors significantly associated with UOA were age, more complex surgery, and longer tourniquet time (*p* < 0.10 all). However, the multivariate analysis revealed that longer tourniquet time was the only significant predictor for UOA (OR = 1.045, 95% CI = 1.022–1.067, *p* = 0.0001). The optimal cut-off points of tourniquet time for predicting UOA with the highest Youden index in the less invasive and more complex groups were 56 minutes and 107 minutes, respectively.

**Conclusion:**

The UOA after AKAS is more common in more complex surgery compared to less invasive surgery. This study showed that unplanned admission significantly associated with many factors—as patient factors, surgical invasiveness, and tourniquet time. However, tourniquet time is the only independent predictor for UOA. Therefore, strict perioperative management protocol must be applied in AKAS, and all patients having these risk factors should be prepared for UOA.

## Introduction

Ambulatory knee arthroscopic surgery (AKAS) is one of the most common minimally invasive orthopaedic surgical procedures and increasingly performed overtime [1] due to the numerous advantages (e.g., the treatment cost with unchanged or improved clinical outcomes) compared to inpatient setting [2, 3]. Nevertheless, previous studies showed that some patients who underwent AKAS still do not experience the expected post-operative recovery and require overnight stay with an incidence of the unplanned overnight admission (UOA) ranged from 0.0004 to 20% [4–6], and the most common cause for UOA was severe post-operative pain [6], while previous studies mostly focused on the factors associated with readmission after AKAS especially in an anterior cruciate ligament (ACL) reconstruction [7–9]. To the best of our knowledge, only few studies explored the factors related to UOA and how to predict UOA after AKAS [10, 11]. Moreover, there were some variations on the peri-operative protocol that might affect the UOA too such as the anaesthetic technique [5], the use of tourniquet and setting pressure [12, 13], and the standardization of operative procedures [4]. Therefore, the aim of this study was to evaluate the effectiveness of a standard peri-operative protocol on UOA and identify the correlation between the peri-operative surgical factors and UOA in patients undergoing AKAS. We hypothesized that some peri-operative surgical factors such as surgical invasiveness and prolong tourniquet time increase risk of UOA after AKAS.

## Patients and methods

### Study design, inclusion, and exclusion criteria

This study was designed as a single-centered prospective cohort study in a medical university hospital and was approved by our institutional review board (protocol number: ID 09–60-07). Informed consent was obtained from all patients who participated in the study, before the surgery was scheduled, in accordance with the Declaration of Helsinki. The manuscript was prepared according to the Strengthening the Reporting of Observational Studies in Epidemiology (STROBE) guideline [14].

The AKAS was developed by a multidisciplinary team comprising sports medicine surgeons, regional anesthesiologists, nurses, and clinical administrators. All patients and their caregivers received pre-operative education and counseling for perioperative instructions and post-operative rehabilitation using instructional video and brochures. The inclusion criteria were the patients who (1) were aged between 18 and 60 years, (2) could follow peri-operative protocol, (3) had the American Society of Anesthesiologists (ASA) physical status grade 1 or 2, (4) had no bleeding disorder, (5) were living around Bangkok or its suburbs, and (6) were willing to participate in this study and give informed consent. The exclusion criteria focused on patients who (1) were unable to communicate due to a physical condition (e.g., hearing loss) or underlying disease (e.g., dementia), (2) had a pre-operative diagnosis of radicular pain or neuropathic pain on the operated knee, and (3) refused to participate.

**Fig. 1:**
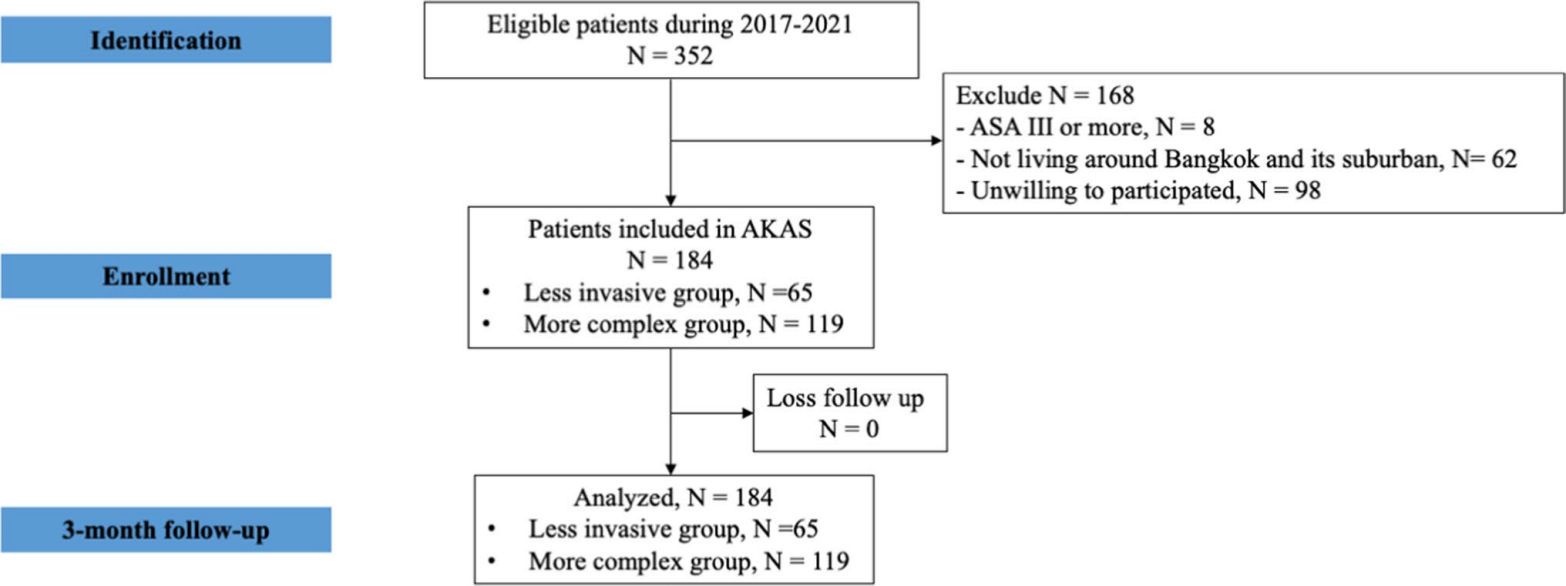
Flow diagram of this study. AKAS, ambulatory knee arthroscopic surgery

Therefore, a total of 184 patients who underwent AKAS at Ramathibodi Hospital between October 2017 and March 2021 were enrolled in this study (Fig. 1). Demographic data are shown in Table 1. Among these patients, the average patient age was 33.4 years (range 15–62 years), and 143 patients (77.7%) were male. The average BMI and pre-operative IKDC score were 25.1 kg/m^2^ (range 16.6–34.6 kg/m^2^) and 52.1 (range 24–97), respectively. The operations were categorized as either less invasive (soft tissue-based surgery) (65 cases, 35.3%) or more complex surgery (ligamentous and meniscal-based surgery) (119 cases, 64.7%). The less invasive group comprised meniscus-alone surgery (51 cases) and other soft tissue surgeries (14 cases), which included arthroscopic debridement, loose bodies removal (8 cases), plica resection (2 cases), excision pigmented villonodular synovitis (2 cases), and implant removal (2 cases). The more complex group comprised ACL reconstruction alone (28 cases), ACL reconstruction with meniscal surgery (90 cases), and medial patellofemoral ligament reconstruction with lateral retinacular release (1 case). The median tourniquet time was 93 min (range 20–163 min). After AKAS, 20 cases (10.9%) were admitted unexpectedly due to NRS ≥ 4 (17 cases), PONV (1 case), NRS ≥ 4 and severe dizziness (1 case), and need of re-operation due to unexpected retained and broken metal guide (1 case). There were no readmissions during the three month post-operative follow-up period.

**Table 1:** Demographic data of 184 patients who underwent ambulatory arthroscopic knee surgery

Demographic data	Total (*n* = 184)
Age, year^◉^	33.4 ± 11.5
Male:female^❖^	143:41
BMI, kg/m^2^^◉^	25.1 ± 3.5
ASA classification grade 1:2	123:61
Pre-operative IKDC score^◉^	52.1 ± 14.0
Operation^☐^	
Meniscus Surgery	51 (27.7)
ACLR alone	29 (15.8)
ACLR with meniscus surgery	90 (48.9)
Other	14 (7.6)
Operation category	
Less invasive:more complex	65:119
Tourniquet time, minute^∆^	93 (20–163)
Unplanned admission^☐^	20 (10.9)
Intolerated pain with NRS ≥ 4	18^a^
Significant PONV	1
Severe dizziness and unable to walk	1
Reoperation	1

*BMI* body mass index, *ASA* American Society of Anesthesiologist, *IKDC* International Knee Documentation Committee, *ACLR* anterior cruciate ligament reconstruction, *NRS* numeric rating scale, *PONV* postoperative nausea or vomiting

^◉^Value presented as mean ± standard deviation

^❖^Value presented as ratio between cases having that condition

^∆^Value presented as median (range)

^☐^Value presented as number of cases (percentage)

^a^One case admitted from concomitant intolerated pain and severe dizziness

### Standard peri-operative protocol

All arthroscopic surgery was randomly performed by six experienced surgeons using the same arthroscopic surgical approach under pneumatic tourniquet 350 mmHg of pressure. All anaesthesia was administered under the care of two anesthesiologists (VA and LS) using the same protocol as combined ultrasound-guided adductor canal block (ACB) and general anesthesia. All patients were first sedated with 0.03 mg/kg of midazolam and 0.5–1.0 mcg/kg of fentanyl before the anaesthetic procedure. The local anaesthetic for ACB was 15–20 mL of 0.5% bupivacaine. Standardized general anaesthesia was initiated with 1–2 mg/kg of propofol and 50–100 mcg of fentanyl intravenously with or without muscle relaxant. Then, a laryngeal mask airway or endotracheal tube was inserted, and anesthesia was maintained with 0.8–1.0 MAC of sevoflurane or desflurane using an oxygen/air mixture. During operation, additional intravenous 0.1–0.2 mg/kg of morphine (maximum 10 mg), 5–10 mg dexamethasone, 30 mg ketorolac or 40 mg parecoxib, and 4–8 mg of ondansetron were given.

### Criteria for discharge

After the completed operation, all patients were subsequently transferred to the post-anaesthetic care unit (PACU) for at least one hour until showing a modified Aldrete score of ≥ 9 [15] and then transferred to a day surgery unit. Additional analgesics (2–4 mg intravenous morphine and 0.5–1.0 g oral acetaminophen) and antiemetics (intravenous 2–4 mg ondansetron or 5–10 mg metoclopramide) were given if the patients had a 10-point numeric rating scale (NRS) of ≥ 4 or post-operative nausea or vomiting (PONV), respectively. All patients were assessed by one of the authors (RW) at six hours after surgery for appropriate discharge. The discharge criteria were as follows: (1) NRS ≤ 3, (2) no PONV, (3) able to independently walk with crutches, (4) no urinary retention, (5) no abnormal surgical wound bleeding, and (6) no re-operation required for any cause. The patients who did not meet the discharge criteria were admitted into the orthopaedic ward for further treatment. All patients were followed at 24 and 48 hours post-operatively by telephone and at two weeks, six weeks, and three months after surgery at the sports medicine clinic or via a telephone interview for clinical evaluation.

### Data collection and outcome measurement

Demographic data included the following: age, gender, body mass index (BMI), ASA physical status, diagnosis, and pre-operative International Knee Documentation Committee (IKDC) score. The peri-operative and post-operative data such as operation, surgical invasiveness category and tourniquet time, discharge status, incidence of UOA, and cause of UOA were recorded. The surgical category was defined as either a less invasive and more complex surgical procedure, based on the surgical invasiveness of outpatient knee surgery, following the prior study by Williams et al. [5, 16] (Table 2). The cause of UOA was defined as any patient problems that did not meet the discharge criteria.

**Table 2:** Categorization of the ambulatory arthroscopic knee surgery based on the surgical invasiveness

	Categories of surgical invasiveness	
	Less invasive	More complex
Example of cases	Knee arthroscopy with:	Knee arthroscopy with:
	Debridement	ACL reconstruction and/or other ligament reconstruction (PCL, MCL, LCL)
	Synovectomy	Posterior oblique ligament or posterolateral corner reconstruction
	Loose bodies removal	Meniscal reconstruction
	Plica resection	Extensive meniscal repair requiring extracapsular suturing
	Meniscal surgery	Proximal/distal patellar realignment
	Meniscal repair	Removal of deeply imbedded hardware
	Removal of superficial hardware	

*ACL* anterior cruciate ligament, *PCL* posterior cruciate ligament, *MCL* medial collateral ligament, *LCL* lateral collateral ligament

### Statistical analysis

STATA SE version 16.0 (StataCorp, College Station, TX, USA) was used to analyze data. Categorical data were analyzed by the Fisher exact test or chi-square test, and continuous variables were analyzed using unpaired *t*-test or Wilcoxon rank-sum test for data as appropriate. Univariate logistic regression analysis was used to determine the predictors associated with UOA. Multivariate logistic regression analysis with stepwise was performed and included variables with a *p*-value less than 0.10 from the univariate analysis. Significant difference was defined as *p*-value < 0.05. The Spearman coefficient and scatter plot were used to identify the correlation between the significant variable and the most common cause of unplanned admission. The receiver operating characteristic (ROC) curve with the Youden index was used to determine the cut-off value for the significant variable. The performance test of the prediction model was calculated to determine the sensitivity, specificity, and positive and negative predictive value in each model.

## Results

### Comparison of perioperative surgical factors between less invasive and more complex surgery

Table 3 shows the data comparison between the patients who had been treated with less invasive surgery (*n* = 65) and more complex surgery (*n* = 119). The incidence of UOA in the more complex group (17 cases, 14.3%) was significantly higher than among those in the less invasive group (3 cases, 4.6%) (*p* = 0.049). The more complex group also showed a significantly higher age and male proportion, lower BMI and pre-operative IKDC, and longer tourniquet time, compared to the less invasive group (*p* < 0.01 all).

**Table 3:** Data comparison between less invasive and more complex surgery

Demographic data	Less invasive group (*n* = 65)	More complex group (*n* = 119)	*p*-value
Age, year^◉^	39.5 ± 13.7	30.1 ± 8.4	< 0.001*
Male:female^❖^	34:28	106:13	< 0.001*
BMI, kg/m2^◉^	25.5 ± 3.8	24.9 ± 3.3	0.228
ASA classification grade 1:2	30:32	91:28	0.0003*
Preoperative IKDC score^◉^	53.4 ± 15.5	51.4 ± 13.1	0.346
Tourniquet time, minute^◉^	47 (20–130)	103 (20–163)	< 0.001*
Unplanned admission^☐^	3 (4.6)	17 (14.3)	0.049*

*BMI* body mass index, *ASA* American Society of Anesthesiologist, *IKDC* International Knee Documentation Committee

^◉^Value presented as mean ± standard deviation or median (range) and compared with unpaired t-test or Mann–Whitney *U* test

^❖^Value presented as ratio between cases having that condition and compared with Fisher exact test or chi-square test

^☐^Value presented as number of cases (percentage) and compared with Fisher exact test or chi-square test

^*^Significant difference with *p*-value < 0.05

### Risk factors for unplanned admission and correlation with the most common cause of admission

Table 4 demonstrates the univariate and multivariate logistic regression analysis for UOA. Based on univariate analysis, the peri-operative factors significantly associated with UOA were age, more complex surgery, and tourniquet time (*p* < 0.10 all). However, multivariate regression analysis revealed that tourniquet time was the only significant predictor for UOA (odds ratio (OR) = 1.045, 95% confidence interval (CI) = 1.022 to 1.067, *p* = 0.0001). The area under the curve of this prediction model was 0.798 (95% CI = 0.733 to 0.854). The Spearman coefficient and scatter plot showed fair correlation [17] between NRS score at 6 h and tourniquet time (*rs* = 0.364, *p* < 0.0001) (Fig. 1).

**Table 4:** Multivariate logistic regression, unplanned admissions (*n* = 184) for ambulatory arthroscopic knee surgery

Variables	UVA			MVA		
	OR	(95% CI)	*p*-value	OR	(95% CI)	*p*-value
Age	0.947	0.900 to 0.996	0.033^p^			
Female gender	0.585	0.163 to 2.104	0.412			
BMI	1.020	0.893 to 1.166	0.768			
ASA grade 2	0.643	0.222 to 1.860	0.415			
Pre-operative IKDC score	0.986	0.952 to 1.021	0.422			
More complex surgery category	3.444	0.970 to 12.232	0.056^p^			
Tourniquet time	1.045	1.022 to 1.067	0.0001^p^	1.045	1.022 to 1.067	0.0001*

^p^Predictors used for multivariate regression analysis with *p*-value < 0.10 in univariate analysis

^*^Significant difference with *p*-value < 0.05

### ROC analysis

The results of using ROC analysis to predict the UOA using tourniquet time are detailed in Table 5. The optimal cut-off point of tourniquet time from this prospective cohort study (*n* = 184) with the highest Youden index was 103 min (Youden index = 0.476, sensitivity 75.0%, specificity 72.6%, PPV 25.0%, and NPV 96.0%). Regarding the surgical invasiveness, for those in the less invasive group (*n* = 65), the optimal cut-off point for the tourniquet time with the highest Youden index was 56 minutes (Youden index = 0.726, sensitivity 100.0%, specificity 72.6%, PPV 15.0%, and NPV 100.0%). For the more complex surgery (*n* = 119), the optimal cut-off point for the tourniquet time with the highest Youden index was 107 minutes (Youden index = 0.422, sensitivity 76.5%, specificity 65.7%, PPV 27.1%, and NPV 94.4%).

**Table 5:** Optimal tourniquet time cut-off points for predicting unplanned overnight admission in whole cohort (*n* = 184), less invasive (*n* = 65), and more complex (*n* = 119) groups

Group	TT cut-off points (minute)	Sensitivity*	Specificity*	PPV*	NPV*
Whole cohort (*n* = 184)	97^a^	80.0 (56.3–94.3)	65.9 (58.1–73.1)	22.2 (13.3–33.6)	96.4 (91.1–99.0)
	103^b^	75.0 (50.9–91.3)	72.6 (65.1–79.2)	25.0 (14.7–37.9)	96.0 (90.8–98.7)
Less invasive group (*n* = 65)	56^a^^,b^	100.0 (29.2–100.0)	72.6 (59.8–83.1)	15.0 (3.2–37.9)	100.0 (92.1–100.0)
More complex group (*n* = 119)	103^a^	82.4 (56.6–96.2)	57.8 (47.7–67.6)	24.6 (14.1–37.8)	95.2 (86.5–99.0)
	107^b^	76.5 (50.1–93.2)	65.7 (55.6–74.8)	27.1 (15.3–41.8)	94.4 (86.2–98.4)

*TT* tourniquet time, *PPV* positive predictive value, *NPV* negative predictive value

^*^Data presented as value in percentage (95% confidence interval)

^a^The cut-off point of tourniquet time yielding at least 80% sensitivity

^b^The cut-off point of tourniquet time with the highest Youden index

## Discussion

Unplanned overnight admission (UOA) has become one of the common peri-operative complications after ambulatory knee arthroscopic surgery (AKAS) and only few studies reported the correlation between peri-operative surgical factors and UOA. This study aimed to evaluate the effectiveness of standard peri-operative AKAS protocol on UOA and identify the risk factors of UOA. Overall, the results from this study showed that the incidence of UOA from AKAS was as high as 10.9% (4.6% in less invasive group and 14.3% in more complex group) with the incidence of readmission at 0% and post-operative pain as the most common UOA cause (Table 2). Moreover, we also found that, with multivariate logistic regression analysis, tourniquet time was a significant independent predictor for the UOA in this study (Table 4).

However, certain limitations in the present study should be acknowledged. First, although this study was designed as a prospective observational study with a standardization of peri-operative protocol, our sample size was relatively small due to the COVID-19 situation. However, our results could demonstrate the significant effect of surgical invasiveness in patients undergoing AKAS and potentially reveal the statistically significant independent predictor of UOA by multivariate analysis (Tables 3 and 4). Second, the incidence of UOA and the findings in this study might be affected by the study inclusion criteria (as ASA status grades 1–2 and the absence of a bleeding disorder) and some uncommon potential factors for UOA (e.g., chronic disease, morbid obesity, and smoking status [10, 11, 18]). Lastly, our results may not be directly applicable in the other conditions related to the use of tourniquet, such as arthroscopic knee surgery without tourniquet inflation [13] and the setting of tourniquet pressure based on limb occlusion pressure (this study used only a 350-mmHg tourniquet pressure setting protocol) [12]. Therefore, future studies should employ a larger sample size, and multicentered prospective studies with different surgical protocol are still required to explore the other predicting factors and benefits of the tourniquet setting protocol.

In terms of the effectiveness of standard peri-operative AKAS protocol, our findings showed that the incidence of UOA are comparable to the results from previous studies [4, 5]. In addition, the present study also demonstrated a significant difference in the characteristics of patients who underwent the AKAS based on the invasiveness of the operations (Table 3), which was also comparable with the results in the previous study by Saltzman et al. [19] where there was significant difference in the demographic data (e.g., age, gender, and BMI) between the patients who received different arthroscopic surgery. Moreover, our results also showed that the patients’ characteristics in more complex group were significantly higher in age and proportion of male gender, higher in ASA physical status, longer in tourniquet time, and greater in UOA, compared to those in the less invasive group. These results are comparable with the previous study by Williams et al. [5] as the UOA in the more complex surgery group was significantly higher than for those who underwent less invasive surgery.

Regarding the multivariate analysis, our results showed that tourniquet time was a significant independent predictor for the UOA (Table 4). Prolonged tourniquet time was associated with a 1.045-fold greater risk of UOA (*p* = 0.0001). The prediction model also demonstrated that the cut-off points for tourniquet time in the whole cohort, less invasive group, and more complex group (103 min, 56 min, and 107 min, respectively) could predict the UOA with a sensitivity of 75.0%, 100.0%, and 76.5%, respectively. These findings imply that longer tourniquet time increases the risk of severe post-operative pain and the risk of UOA. Such implications align with results in previous studies by Lutz et al. [20] and Boddapati et al. [10] that prolonged tourniquet time of more than 50 minutes was one risk factor of severe post-operative pain (NRS ≥ 7) [20] and that procedures longer than 90 minutes are a risk factor for UOA [10]. Moreover, the present study revealed that the cut-off point for the AKAS procedure could vary between less invasive and more complex surgery, and the cut-off time for these different procedures should be based on the level of surgical invasiveness. Therefore, to achieve the optimal outcome, we recommended that strict peri-operative management protocol during AKAS must be applied in all patients. Also, the patients, who have those risk factors and are expected to have prolonged tourniquet time, should be informed for the risk of UOA, closely monitored for the post-operative complication, and prepared for overnight admission.

## Conclusion

This study showed that the incidence of UOA after AKAS could be as high as 10.9% and that the most common cause of UOA was pain-related. Moreover, many factors such as patient factors, surgical invasiveness, and tourniquet time were significantly associated with unplanned admissions. However, tourniquet time was the only independent predictor for UOA with the cut-off point of 107 minutes.

## Data Availability

The datasets generated and/or analyzed during the current study are available from the corresponding author upon any reasonable request.

## Abbreviations

ACB: Adductor canal block
ACL: Anterior cruciate ligament
AKAS: Ambulatory knee arthroscopic surgery
BMI: Body mass index
CI: Confidence interval
IKDC: International Knee Documentation Committee
NPV: Negative predictive value
NRS: Numeric rating scale
OR: Odds ratio
PONV: Post-operative nausea or vomiting
PPV: Positive predictive value
ROC: Receiver operating characteristic
UOA: Unplanned overnight admission
